# Is composition of vertebrates an indicator of the prevalence of tick-borne pathogens?

**DOI:** 10.1080/20008686.2022.2025647

**Published:** 2022-01-10

**Authors:** Agustín Estrada-Peña, Natalia Fernández-Ruiz

**Affiliations:** a Department of Animal Health. Faculty of Veterinary Medicine, University of Zaragoza, Zaragoza, Spain; b Instituto Agroalimentario de Aragón (Ia2), Zaragoza, Spain

**Keywords:** Keystone communities, vertebrates, Europe, tick-borne pathogens, indicator

## Abstract

Communities of vertebrates tend to appear together under similar ranges of environmental features. This study explores whether an explicit combination of vertebrates and their contact rates with a tick vector might constitute an indicator of the prevalence of a pathogen in the quest for ticks at the western Palearctic scale. We asked how ‘indicator’ communities could be ‘markers’ of the actual infection rates of the tick in the field of two species of *Borrelia* (a bacterium transmitted by the tick *Ixodes ricinus)*. We approached an unsupervised classification of the territory to obtain clusters on the grounds of abundance of each vertebrate and contact rates with the tick. Statistical models based on Neural Networks, Random Forest, Gradient Boosting, and AdaBoost were detect the best correlation between communities’ composition and the prevalence of *Borrelia afzelii* and *Borrelia gariniii* in questing ticks. Both Gradient Boosting and AdaBoost produced the best results, predicting tick infection rates from the indicator communities. A ranking algorithm demonstrated that the prevalence of these bacteria in the tick is correlated with indicator communities of vertebrates on sites selected as a proof-of-concept. We acknowledge that our findings are supported by statistical outcomes, but they provide consistency for a framework that should be deeper explored at the large scale.

## Introduction

Communities are sets of organisms that tend to appear together in ecosystems, from microbes [i.e. 1] to higher vertebrates or plants. Members of a community interact and can colonize a range of environmental conditions, with species exhibiting different relative abundances. Interactions among species provide a view of the community in equilibrium; this is the ‘optimal composition and size’ of a community under a given set of (a)biotic constraints. Other than the use of communities of organisms as bioindicators of the health of ecosystems [2], they can be used to assess the quality of human health and the predicted future response to changes in climate [3]. Hawkins et al. [4] stated that the determinants of local biodiversity and variation of organisms are a central aim of modern ecology, and that a basic set of temperature, rainfall, or evapotranspiration ‘can account for much of the variation in plant and animal species diversity across space.’ [4].

It has been recognized that parasites influence host communities in different ways [5,6] but the opposite view, i.e. how the vertebrates’ community affects parasites, has been less addressed [i.e., 7, 8]. There are excellent examples, generally performed on the regional scale, about the impact of the community structure of vertebrates on the faunal composition of parasites [9,10]. Regarding the effects of vertebrates’ communities on the circulation of vector-borne pathogens, it has been demonstrated that the relative composition of communities may influence the patterns of tick-borne pathogen infection rates [summarized by 11]. As an example, it has been pointed out that some carnivores may play only a small role in the circulation of the tick-borne pathogen *Borrelia* spp, but they are able to influence the density of small mammals and birds by a predator–prey cascade of effects [13] on the rodents and birds that are reservoirs of the pathogen. The composition of the vertebrates affects the entire complex of vertebrates-ticks-pathogens [14,15].

The issue surrounding tick-borne pathogens revolves around the questions of: (i) whether there is a combination of vertebrates behind the range of existing field observations of prevalence of a tick-borne pathogen, (ii) whether it is a reproducible finding for each ecosystem, and (iii) how the alterations of these natural communities may affect the circulation of the tick(s) or the pathogen(s). There are obvious logistical issues in conducting large-scale tick surveys [14]. By way of example, a large meta-study was carried out on the reservoir capacities of some well-studied reservoirs of tick-borne bacteria of the genus *Borrelia*, correlating the abundance of *I. ricinus* and the prevalence of *B. burgdorferi* s.l. with morphological and physiological traits of the animals, concluding that ‘few vertebrate species dominate the *B. burgdorferi* s.l. life cycle’ [16]. Other studies have conducted research on the significance of different vertebrates in the transmission rates of tick-borne pathogens [i.e. 17, 18].

A pending issue in this field of research is the translation of an explicit composition of vertebrates into an epidemiologically coherent indicator for the circulation of a tick-borne pathogen on a large scale. It must be noted that some vertebrates just feed the tick, while others contribute to the circulation of pathogens (acting as reservoirs). The rate of ticks feeding on reservoirs is a spatially variable feature generated by the community of available vertebrates that could dramatically change the prevalence of a pathogen in ticks. It depends upon the relative abundance of each vertebrate and its ‘attractiveness’ to the tick, plus the status of the vertebrate as a reservoir or not. The idea of the importance of vertebrates’ communities on the complex patterns of dilution or amplification of prevalence of *B. burgdorferi* s.l. in ticks has been discussed and summarized by 19. These authors enumerated the issues regarding the impact of vertebrates’ composition on the infection rates of tick-borne pathogens and wrote ‘combined with data on host feeding utilization, infection prevalence and duration and magnitude of infectiousness [the data on hosts and reservoirs] could be used to make predictions of nymphal infection prevalence across space or time.’

The impact of the joint contribution of a community of vertebrates on the prevalence of a tick-borne pathogen has been systematically neglected for large regions. The relative composition of a community of vertebrates changes along spatial scales as a response to gradients of environmental conditions that also impact contact rates with ticks. Knowledge of the effects of the faunal composition of vertebrates across a geographical gradient on tick-borne pathogens would provide an unparalleled framework, helping to evaluate the relative importance of vertebrates’ composition on the circulation of tick-borne pathogens. Such a description of an ‘indicator community’ could then be expected to describe the prevalence of a pathogen in questing ticks.

This study aims first to classify the territory of the western Palearctic into clusters, using the known distribution of 165 species of vertebrates reported as hosts for the tick *I. ricinus*, in a method known as bioregionalization. Each cluster results from similar presence and abundance patterns of a set of vertebrate’s species, statistically different from those found in other clusters. We calculated both the species richness and the phylogenetic diversity of the communities per cluster. We further correlated the composition of the communities against the prevalence of either *Borrelia afzelii* or *Borrelia garinii*, two major pathogens transmitted by that tick, as a proof-of-concept, using data about questing nymphs of *I. ricinus* at the European scale. The purpose is not to predict infection rates in ticks in the territory, but to demonstrate that an indicator community exists, correlating with the prevalence of a tick-borne pathogen even on a large scale. The main novelty of our approach is the building of spatial regions according to the vertebrates’ composition, thus proposing a method to de-correlate the animals’ assemblages with the abiotic factors.

## Material and methods

### Background

Following the concepts provided by [20], we refer to the exercise of obtaining spatial units with a similar composition of vertebrates as ‘bioregionalization’. It is a classification technique. This study focuses on the characterization of clusters that have a similar composition of vertebrates in the western Palearctic, weighted by contact rates with the tick *I. ricinus* [21]. As a proof-of-concept, we outlined the indicator community in selected clusters of the target region against the infection rate of two species of *B. burgdorferi* s.l. in questing *I. ricinus* nymphs, as previously compiled and reported [22].

### *Compiling the reported distribution of vertebrates and* I. ricinus *in the target territory*

We collected information about the recorded distribution of the vertebrate hosts for *I. ricinus* in Europe, with coordinates, originally published by Estrada-Peña and de la Fuente (2016) (data available at https://datadryad.org/stash/dataset/doi:10.5061/dryad.2h3f2). These data produced the maps of the predicted distribution of vertebrates, also available in the same link. We updated the outcome of that previous analysis only for the species of vertebrates for which the number of records reported had increased by more than 10% since the date of the previous publication (2016) using new records from GBIF (https://www.gbif.org, last accessed March 2020). Since the proof-of-concept of this study uses the distribution of the tick *I. ricinus*, vertebrates were selected to include only those species hosting the tick (166 species reported). We acknowledge that this does not reflect the complete distribution of *all* the vertebrates in the western Palearctic, but rather, those that have an impact on the circulation of the selected pathogens. In total, we handled more than 3 million geo-referenced records of vertebrates and more than 14,000 records of *I. ricinus*.

### Mapping the distribution of vertebrates

For calculations of the predicted distribution of both the tick and vertebrates recorded as hosts or reservoirs of *Borrelia,* we used a series of monthly values of temperature, soil humidity, and water vapor deficit between the years 1980 and 2018, from the TerraClimate repository (http://www.climatologylab.org/terraclimate.html, last accessed March 2020). The complete time series was summarized as the monthly average of each variable. Each set of average monthly values was subjected to harmonic regression. The use of harmonic regression coefficients has been previously validated [23] since they are free of the frequent issues of spatial correlation and multicollinearity between layers. Harmonic regression produces the best fit for seasonal variability of each variable, and each regression curve has several coefficients. We used the first three coefficients of the harmonic regression for each climate variable as explanatory variables for predictive mapping (total: 9 explanatory variables).

We independently modeled the presence of each species using the niche modeling algorithm MaxEnt integrated in the ‘dismo’ package [24] for R [25]. Models were developed with linear and quadratic features, using a variable number of background points (10,000–100,000), 10 replicates per species were modeled, and 70% of points for training purposes. We used cross-validation to compare the resulting models. The variable number of background points was proportional to the number of actual records. This strategy was implemented because some species have many records in the target region, while others are poorly represented. Each model was replicated 100 times, partitioning the data into replicate folds, with each fold used in turn to test the model. The regularization multiplier was set to 1.

We evaluated the performance of the models using the Boyce index implemented in the ‘ecospat’ package 26 for R [25]. We did not use the usual index of the Area Under the Curve (AUC) since it has received criticism as being affected by background area. Considering that we are dealing with vertebrates that may have a relatively restricted distribution, the ratio between the size of the background and the actual vertebrate’s distribution may have an impact on modeling [27]. Rotllan-Puig and Traveset 28 commented on the rationale behind the Boyce index that varies between – 1 and +1. The only species of vertebrate that had poor modeling values according to the Boyce index was *Luscinia luscinia*, which was dropped from the final dataset, resulting in the total of 165 distribution maps. However, this new modeling exercise did not change the original conclusions on the predicted distribution (maximum change <1%, recorded for *Capreolus capreolus*; values of change for the remaining species well below the 0.5%); therefore, we continue considering the maps of Estrada-Peña and de la Fuente (2016) as a valid picture of the predicted distribution of vertebrates and *I. ricinus* over the target territory. The final maps intended for bioregionalization (see point 2.4) display the expected distribution of each vertebrate and its environmental suitability (translated here as ‘abundance’).

We then explicitly addressed the contact rates between each vertebrate and *I. ricinus* as reported [21], calculating the overlap of habitat throughout the complete target territory on a pixel-by-pixel basis. This was done in two steps, first using the function ‘pno’ (predicted niche occupancy) in the package ‘phyloclim’ [29] for R, following the concepts by [30], on which the function ‘niche.overlap’ calculates the percent of the environmental niche that is shared by any pair of species. The result represents a measure of the amount of habitat shared by any tick-vertebrate combination [21].

To note, we did not consider the actual contribution of each species of vertebrates to support the feeding of the tick because ‘tick preferences’ to feed on different vertebrates, obtained from field or laboratory data, as summarized by [16]. We think that reliable field data are available for only the most surveyed vertebrates; they are thus unavailable for more than 90% of the species included in this study. Therefore, the inclusion of the empirical data on host’s preferences by the tick for the few available species would introduce a distorting variable because it could not be applied to every pair of associations tick-vertebrate. The tick preferences towards each vertebrate were obtained from two previous reports, derived from the centrality index of a network analysis on published records of *I. ricinus* on hosts [21]; the raw files to build the networks are available at https://datadryad.org/stash/dataset/doi:10.5061/dryad.2h3f2.

### Clustering the distribution of vertebrates into spatial units

A stack consisting of 165 layers of vertebrates’ raster maps was used for an unsupervised classification of the territory. The purpose is to classify the territory into clusters based on the abundance of each vertebrate and the contact rates with the ticks by pixel. We applied an unsupervised classification that used the aforementioned stack of maps but did not supply any response data (that is, we did not identify any pixel as belonging to a particular class). This technique is useful when we have no prior knowledge of the study area. We used the k-means clustering algorithm to process a set of maps that resulted in the bioregionalization. To implement a k-means classification algorithm, the target number of regions (k) was determined by maximizing the cluster validity index. The Calinski–Harabasz Variance Ratio Criterion (VRC) 31 was used to measure within-group and between-group dispersions. The classification produced a set of areas representing a unique combination of vertebrates’ species, their abundance (from modeling), and contact rates with the tick. Clusters of the same category are statistically inseparable, and clusters belonging to different categories are statistically different. The optimal number of categories for the target territory was 36, although two of them were returned as ‘empty clusters’ due either to the absence of vertebrates or of *I. ricinus*.

### Measuring the phylogenetic diversity of the vertebrates

We asked if the phylogenetic diversity of each vertebrates’ community is correlated with certain traits of the tick presence or abundance (modeled). The Open Tree of Life is an online phylogenetic tree of life that is updated by adding published and curated phylogenetic trees of any organism. The project integrates these new trees as they are published into the mega-tree hosted by the website. It is thus possible to query the complete mega-tree and a subset of a number of species or other taxa ranks to prepare an *ad hoc* synthetic tree. These subset trees are not ultrametric, and it is necessary to calculate the branch length between any pair of species to have the phylogenetic measures of interest. We used data from the Open Tree of Life (https://tree.opentreeoflife.org, last accessed March 2021) for phylogenetic calculations, accessing its API using the ‘rotl’ package [32] for R.

We calculated the phylogenetic diversity (PD) and species richness (SR) existing in each cluster of the target territory using the package ‘picante’ [33] for R. While SR is a pure count of species, PD estimates the amount of phylogenetic variability in a cluster using the sum of the lengths of the branches of the phylogenetic tree of vertebrates present in that cluster. For the main calculations, we used only the generic name of each vertebrate to improve the solidness of the outcome. Supplemental material 1 includes the phylogenetic trees of the vertebrates’ species together with the use of these hosts by *I. ricinus* and includes a ‘readme’ file (in PDF format) with information about all the files. Supplemental material 2 includes these same phylogenetic trees of vertebrates’ species together with the spatial context of the target area.

### Proof-of-concept: discriminating the prevalence of Borrelia spp. in questing ticks using the composition of communities

**Figure 1.**
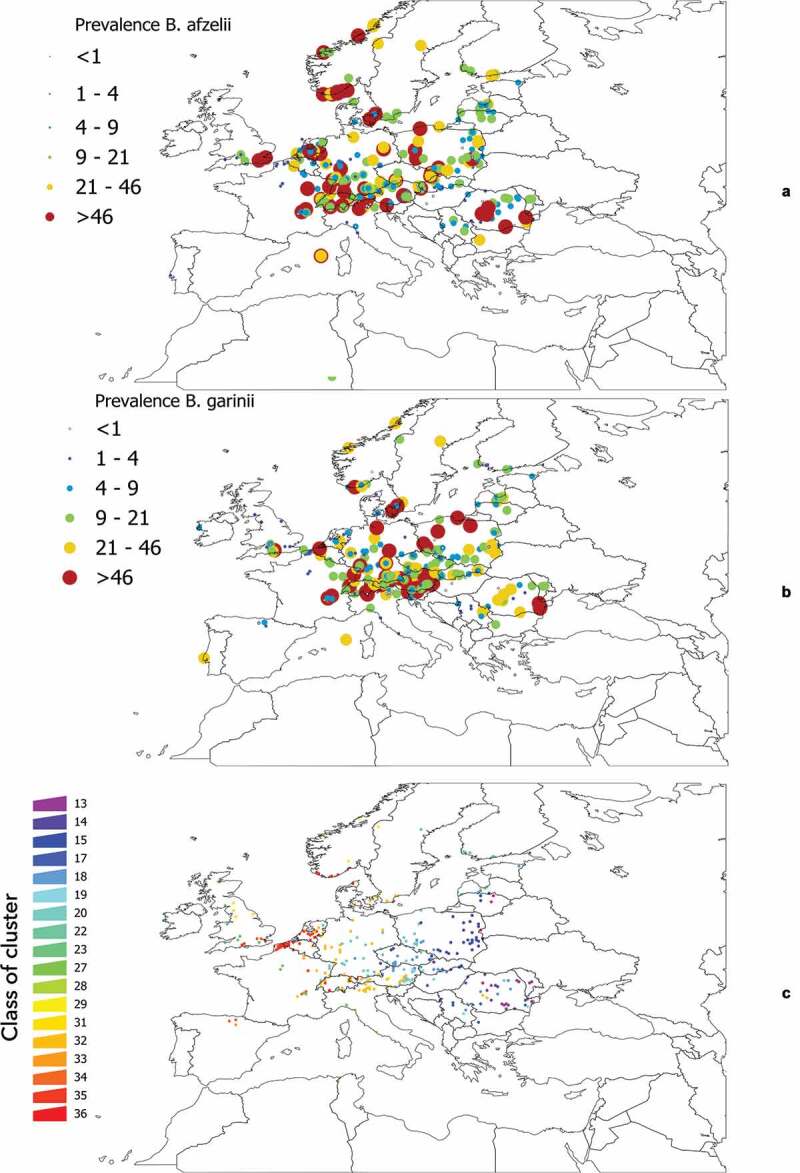
General background of distribution of *Borrelia* spp. in western Palearctic and the individual sets of clusters used for further analyses. a: The coordinates of records of *B. afzelii* in questing nymphal *I. ricinus* as reported in the published literature. b: The coordinates of records of *B. garinii* in questing nymphal ticks as reported in the published literature. For both a and b, compilation finished in the year 2018. Color and size of the dots mean for the reported prevalence. c: The sites used for statistical evaluations between the communities of vertebrates and infection rates of *Borrelia* spp. were calculated. The color of each point (which is actually an hexagon whose diameter is 0.25º) corresponds to the correlative numbering of the clusters obtained from the bioregionalization of vertebrates.

We aimed to demonstrate that, at a rough scale of landscape, there is an indicator community of vertebrates that could describe the infection rates of *B. afzelii* and *B. garinii* in questing ticks. We chose these two bacteria because they are widely distributed in Europe, they have different vertebrate reservoirs (in general terms, birds for *B. garinii*, rodents for *B. afzelii*), and reports point to a role of community composition on the infection rates 34. It is not possible to develop these calculations for the complete target territory (due to lack of data on prevalence in ticks), or to apply the hypothesis to the *points* (coordinates) in which *Borrelia* spp. have been reported. Therefore, we covered the target territory with a hexagonal tessellation with a radius of 0.25º, selecting only those cells in which data of prevalence in ticks of either *B. afzelii* or *B. garinii* (or both) have been published. The choice of the diameter is not unintentional: higher cell sizes blurred the results (many species of vertebrates in the same cell) and smaller cell sizes commonly overfitted the models (too few species of vertebrates present in the cell). Data on the distribution of *Borrelia* spp. in Europe were obtained from [22], accounting for the prevalence of the pathogens in questing ticks. This produced 549 records for *B. garinii*, 555 records for *B. afzelii*, and 319 cells of the different clusters further used for modeling exercises (Figure 1)]. No limitations on the sample size of ticks were introduced as constraints in the selection, aiming to increase the number of sites to test.

The dataset on prevalence rates of *Borrelia* in ticks has been used as published [22]. In short, the dataset was compiled from scientific literature, between the years 1990 and 2017. All the details about the bibliographical query are described in the original study [22]. To summarize, only data from molecular detection of the pathogen(s) on questing nymphs of *I. ricinus* were used. We excluded every report of ticks collected while feeding, since it is not possible to ascertain if the DNA of the pathogen was already in the tick or was acquired with the blood meal. We also excluded field data on larvae or adults, the former because there is no transovarial transmission of these pathogens and the larvae hatch free of them; the latest because adults are more difficult to collect, and surveys tend to produce consistently a fewer number of specimens. As mentioned, only molecular tests (qPCR, RT-PCR) were used; other tests, like dark field microscopy or xenodiagnoses (i.e., infection of naïve hosts allowing ticks collected in the field to feed), while useful in its own context, are not comparable among them, thus biasing the statistical comparisons.

The purpose of modeling is two-fold, namely (i) to explain the prevalence of *Borrelia* spp. in questing for nymphal ticks and (ii) to delineate the indicator community that shapes the recorded infection rates in ticks. Modeling was done in the Orange Programming Environment (which is freely available from https://orangedatamining.com).

#### *Modeling the vertebrates’ communities that might drive the prevalence of* Borrelia *in the vector*

We purposely chose sites of the same cluster category, displaying differences in infection rates with other clusters and with a minimum of 15 independent surveys of questing ticks. We ultimately selected clusters 32 and 20 for *B. afzelii* (average reported prevalence in questing nymphs 10.5% and 22.4%, respectively) and clusters 35 and 20 for *B. garinii* (average reported prevalence in questing nymphs 7% and 15%, respectively). It was difficult to find clusters with lower prevalence of *Borrelia* spp. (i.e., lower than 7%) because the scarcity of these records in published literature (less than seven different surveys) compromising the quality of the statistical outcomes. It was impossible to select a significant number of clusters with a reported prevalence of ‘0.’ The mere lack of reporting at a site (thus prevalence = 0) could mean that surveys have never been conducted at that site.

All algorithms for model development were available in the Orange programming environment. Supplemental material 3 includes the scripts using a graphical interface for repeating the modeling exercises or issuing new ones under different conditions. The ecological meaning of the calculations is also shown as separate charts in the body of the text. We used four different modeling approaches to correlate a given combination of vertebrates with the prevalence of *Borrelia* spp. in questing ticks: (i) Neural Networks, (ii) Random Forest, (iii) Gradient Boosting, and (iv) AdaBoost. All are algorithms of ‘regression and classification’ that operate on numerical data to obtain a response. Neural networks are comprised of node layers, containing an input layer, one or more hidden layers, and an output layer. Each node connects to another with a weight and a threshold. If the output of the node is above the specified threshold value that node is activated, sending data to the next layer of the network. The specific combination of ‘on-off’ nodes provides the solution [35]. For Neural Networks, we used 100 neurons per hidden layer, the ReLu algorithm, the Adam solver and 200 iterations. Random Forest, an ensemble learning method developed by 36, builds a set of decision trees. Each tree is developed from a bootstrap sample from training data. For each individual tree, an arbitrary subset of attributes is drawn from which the best attribute for the split is selected. The final model is based on the majority of votes from individually developed trees in the forest [37]. For Random Forests, we included 10 trees (i.e., the number of decision trees will be included in the forest), five trees to be split, which specifies the number of attributes that are arbitrarily drawn at each node at every step of the tree’s development, without balance of classes, and three replicates of each model. Gradient boosting [38] is a method for creating an ensemble that starts by fitting an initial model (e.g., a tree or linear regression) to the data. A second model is then built, focusing on improving predictions where the first model performs poorly. The combination of these two models is expected to be better than either model alone. The process is then repeated many times, each successive model attempting to correct for the shortcomings of the combined boosted ensemble of all previous models. For Gradient Boosting, we used 100 trees with a learning rate of 0.1. AdaBoost (short for ‘adaptive boosting’) is a machine-learning algorithm, formulated by 39,that uses learning algorithms and iteratively tries to improve the solution in an adaptive way (tweaking weak learners in favor of those instances misclassified by previous classifiers.]. For AdaBoost, we used 50 estimators, a learning rate of 1, the SAMME.R classification algorithm and a linear regression lost function.

#### Detection of indicator communities and infection rates by *Borrelia.*

The modeling algorithms mentioned in the previous point are addressed to predict the prevalence of the pathogen in the questing tick using the abundance and contact rates of the tick vector with the vertebrates. Here, we explicitly asked for the ‘indicator community’: the subset of vertebrates that better explains these infection rates, removing species that have little or no significance in the outcome. Our purpose is not to state the individual roles of each vertebrate, but rather, the effects of the whole community on the observed prevalence of the pathogen(s) in ticks. We used a ‘rank filter’ based on RReliefF [a method originally developed by 40]. The filter employs a stand-alone modeling algorithm to extract the set of candidate subsets of vertebrates that contribute most to the modeling results. In other words, the rank filter extracts variables with the highest impact on the results and promotes them as the ‘best set of vertebrates’ that are behind the observed prevalence of *Borrelia* spp. in questing ticks.

As mentioned, Supplemental data 3 includes the scripts for Orange and the input files necessary for reproducing the complete set of calculations explained in section 2.6 of Methods. Interested readers should have a basic knowledge of Orange programming environment to reproduce the results.

## Results

### *Clusters of vertebrates and* I. ricinus *in western Palearctic follow a gradient of climate*

**Figure 2.**
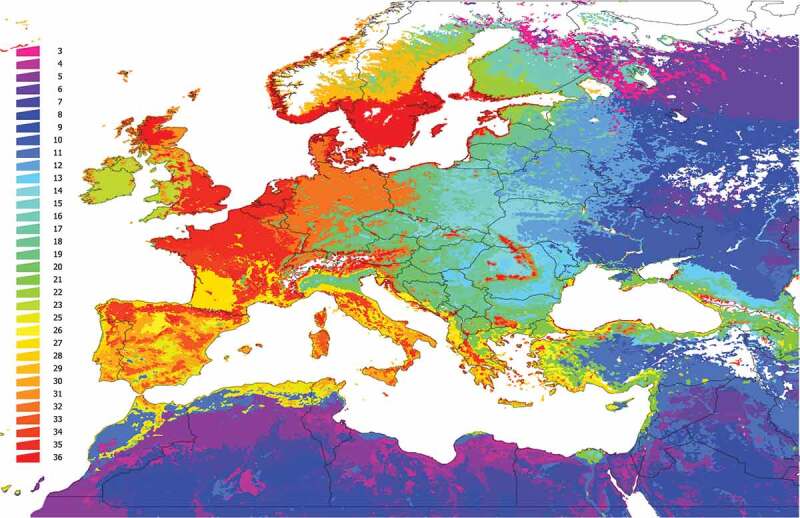
Clustering and the regions resulting from bioregionalization of the expected distribution of vertebrates in the target region, the expected distribution of *Ixodes ricinus* and its niche overlap. The map was obtained using an unsupervised classification using k-means on the raster maps of the predicted distribution of 165 species of vertebrates and *I. ricinus* and calculating the predicted niche occupancy of every pair of combinations vertebrate – tick. The unsupervised classification returned 36 categories, of which the 1 and 2 are in northern Scandinavia, western Russia, and eastern Turkey, where *I. ricinus* is absent. We kept the remaining 34 categories. Colors of the figure are random.

**Figure 3.**
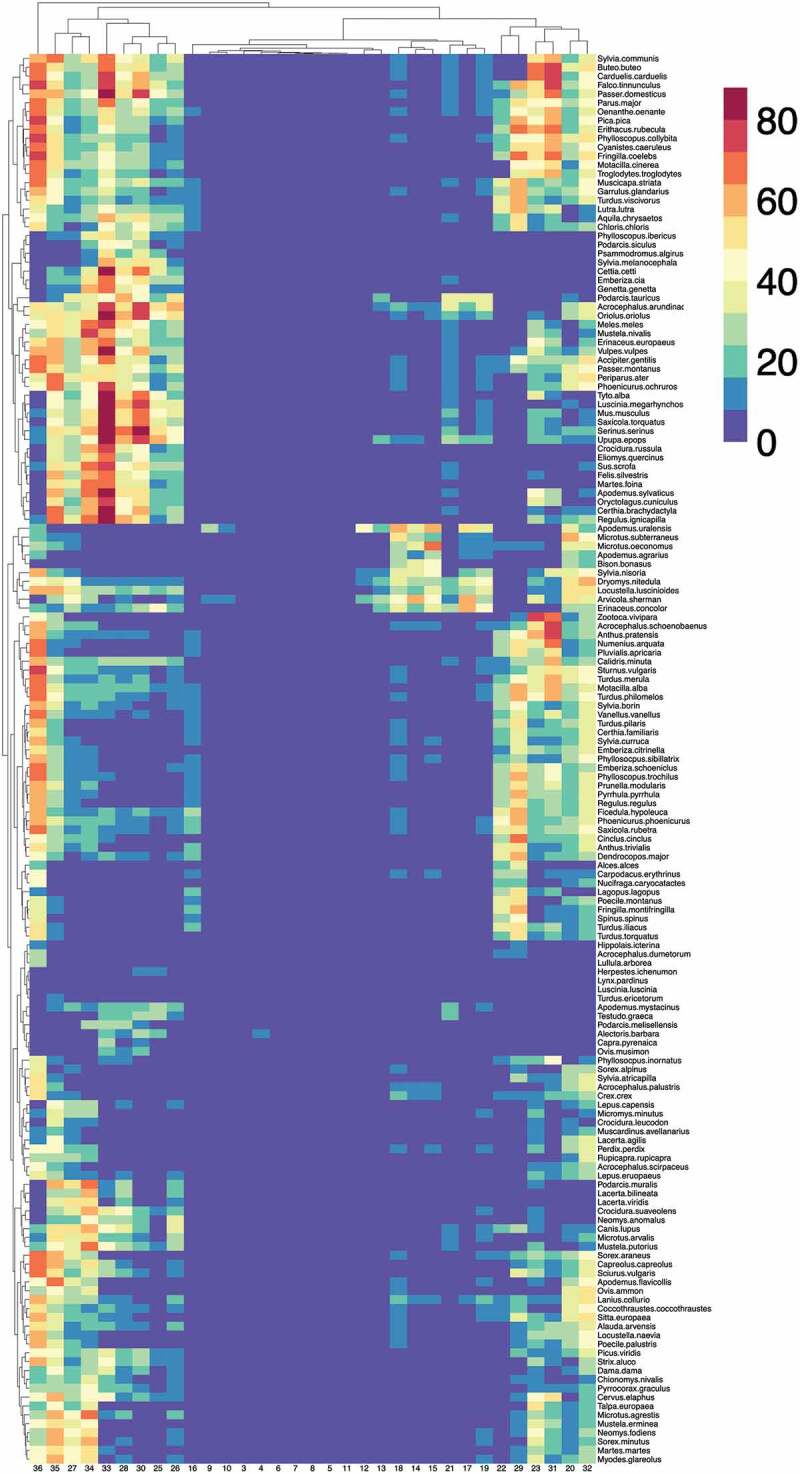
A heatmap representing the abundance of vertebrates, weighted by the contact rates with *Ixodes ricinus* in the western Palearctic at each cluster of the territory. The values in the heatmap show two dendrograms, one for the vertebrates that tend to appear together (left of the figure) and the other for sites that tend to support similar fauna of vertebrates (top of the figure). Specific names for every vertebrate are included, even if the phylogenetic tree of vertebrates has been made using only generic denominations.

The unsupervised classification of the territory using the k-means algorithm produced 36 clusters, shown in Figure 2. Note that clusters are defined by the composition of 165 species of vertebrates and their relative abundance weighted by contact rates with *I. ricinus*. Clusters 1 and 2 were restricted to the coldest mountain regions of Scandinavia and either the vertebrate species targeted in the study or the tick are predicted to be absent for these areas. According to the clustering methods, areas with the same color in Figure 3 have a more similar vertebrate composition/contact rates within them than among other areas. Clusters have similar communities of vertebrates to the level of significance p = 0.05. All clusters depicted in Figure 2 are statistically different from the faunal composition of other clusters in terms of vertebrate communities. Clusters of the same color may be spatially separated by other clusters.

### High contact rates of tick and vertebrates are concentrated in clusters with highest phylogenetic diversity

Figure 3 represents the calculated environmental suitability/contact rates for vertebrates in clusters of the target region. The heatmap also includes two dendrograms: one for vertebrates (linking those that tend to appear together) and one for clusters (linking those that tend to have similar vertebrate composition). Note that there is a clear gradient of spatial variability. Of interest (comparing Figures 2 and 3) is the poor variability of some territories, including mainly desert areas of northern Africa and contiguous Asia, and the higher species diversity in others. These patterns are not only affected by the ‘abundance’ of each vertebrate but also by the contact rates with *I. ricinus*, showing low values in clusters where niche overlap between tick and vertebrates is low. Note that many clusters in the territory (grouped mainly in north, central, and Western Europe) are predicted to carry large fractions of the complete set of vertebrates in this study, suggesting both a substantial contact with *I. ricinus* in these areas.

**Figure 4.**
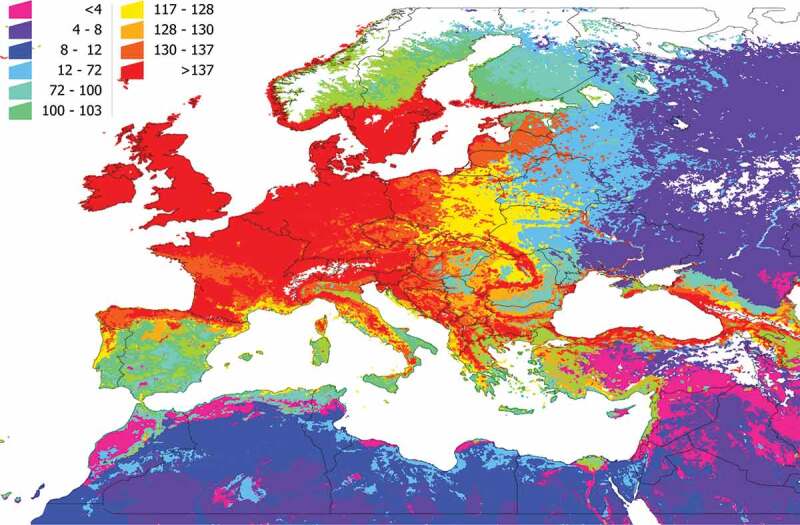
Species richness of vertebrates in the target territory. The value must be interpreted as the number of vertebrates reported as hosts of *I. ricinus* that are expected to be present in the territory and available for the tick because they share portions of the environmental niche.

**Figure 5.**
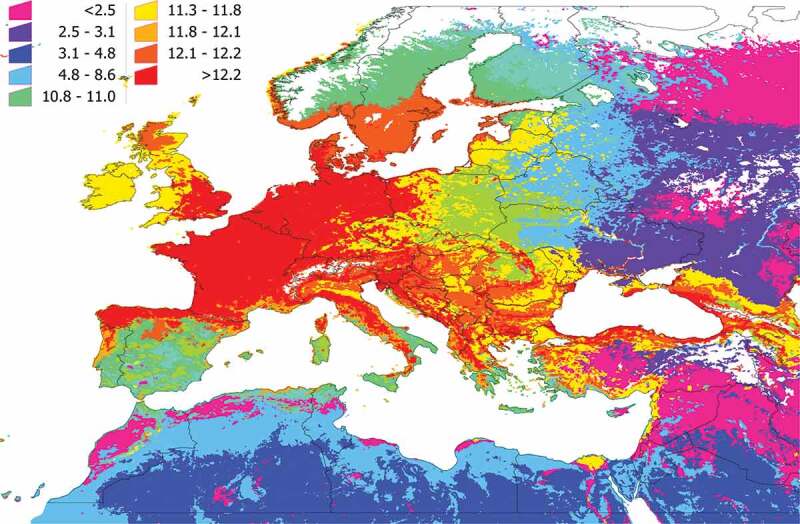
The phylogenetic diversity of the vertebrates in the target territory. The value must be interpreted as the phylogenetic diversity of the vertebrates reported as hosts of *I. ricinus* that are expected to be present in the territory, measured by the method of Faith. Supplementary Research Data contains the details of the spatial distribution of each cluster, and the use of portions of the phylogenetic tree of the vertebrates by the tick in each cluster.

We calculated the PD and SR of each cluster (Figures 4 and 5). The values of both indexes do not perfectly overlap because species richness is not the same as phylogenetic diversity: areas of high SR may have a low PD because existing vertebrates are phylogenetically close. In general terms, most of the western Palearctic has a PD higher than 10 (a value considered high), meaning there is a wide range of potential, *phylogenetically distant* hosts for *I. ricinus*. Most of Central Europe, the Baltic countries, and southern Scandinavia, as well as parts of northern Spain and other mountain ranges (i.e., in Italy or Romania) displayed a high PD, suggesting large communities of phylogenetically separated vertebrates that could interact with the tick. Further on this, the highest environmental suitability for *I. ricinus* overlaps the territories with the highest PD of vertebrates (R^2^: 0.897, F: 1287.14, p < 0.05). Values of SR and PD tend to attenuate in the eastern range of the map and in northern Africa. Since the contact rate of the tick with the vertebrates is part of our strict definition of communities, such attenuation of values should be expected because *I. ricinus* is predicted to be mostly absent from the mentioned regions.

Correlations between the prevalence of *B. afzelii* or *B. garinii* with the PD or SR of each cluster were far from significant (PD: R^2:^ 0.0009 for *B. afzelii* and 0.0012 for *B. garinii*; SR: R^2:^ 0.0014 for *B. afzelii* and 0.0125 for *B. garinii*I; p > 0.8 in both cases) indicating that the prevalence of the pathogens is not only correlated with the contact rates of *I. ricinus* with *any* vertebrate. Therefore, the mere co-existence of large populations of the tick and a high number of available vertebrates, is not a hallmark for the circulation of the chosen pathogens. This suggests that the pathogens could be linked to peculiar combinations of vertebrates feeding the tick.

### Proof-of-concept: detecting the communities driving the prevalence of B. afzelii and B. garinii in selected clusters

We asked whether indicator communities exist as the best index of infection rates by either *B. afzelii* or *B. garinii* in *I. ricinus*. This is not to evaluate whether each cluster resulting from bioregionalization carries a *unique indicator species* of vertebrate shaping high or low values of prevalence of *Borrelia* spp. in questing ticks. The goal is to find communities displaying statistically solid relationships with the patterns of prevalence in ticks. This analysis cannot be done on single sites belonging to a given cluster but rather by using *all* sites belonging to the same category of clusters. With these cautionary words, we first concluded that the infection rates in questing ticks are statistically different among the clusters, as detected by an ANOVA test (*B. afzelii*: F: 77.08; p < 0.0001; *B. garinii*: F: 1042.3; p < 0.0001).

**Figure 6.**
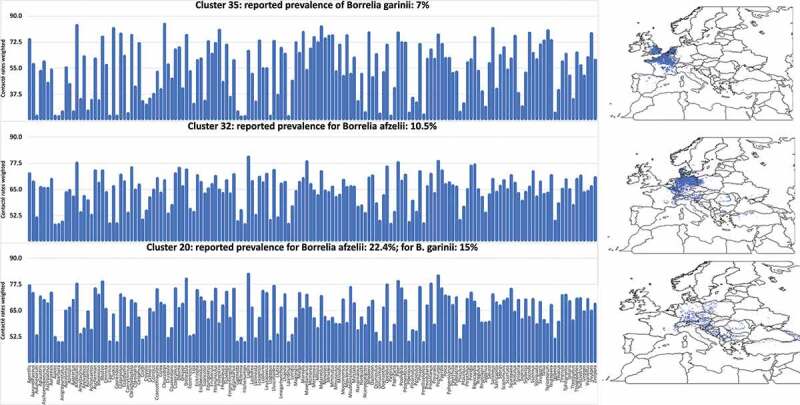
The modelled suitability of each species of vertebrate included in this study, weighted by the contact rates with the tick *I. ricinus* and the preferences of the tick for each host (histograms). Only data for areas included in the categories 20, 32 and 35 are shown, since they represent the most contrasting sites regarding prevalence of *Borrelia* spp. in the target territory. These ar ethe sites that were subjected to modeling. A small map at right shows the spatial extension of these territories. All these data were obtained from Supplemental material 3.

The ecological meaning of the proof-of-concept is schematized in Figure 6. It represents the communities of vertebrates in the three different clusters selected for testing by the modeling algorithms (whose spatial distribution is shown in the accompanying maps) expressing the contact rates with *I. ricinus* corrected by the hosts preferences, and the prevalence of *Borrelia* spp. reported in questing nymphal ticks. At a first view, it is difficult to observe a pattern. The task of the algorithms is twice: (i) evaluate the combinations of vertebrates’ species to obtain a better correlation with tick prevalence, and (ii) deduce a better combination of vertebrates’ species that produces that outcome and display it in a reduced space of principal components. To note, vertebrates ‘compete’ for the tick, and it may have high contact rates with a non-reservoir vertebrate (therefore ‘diluting’ the circulation of the pathogen) or with a prominent reservoir. The modeling must ‘remove the noise’ leaving only the most important vertebrates whose joint contribution describes the field findings.

#### *The performance of the models: explaining the prevalence of* Borrelia *spp. through the vertebrates’ composition.*

The results of the modeling algorithms for each cluster and both *B afzelii* and *B. garinii* are included in Table 1. Several algorithms displayed high reliability, accounting for the effect of the combination of some species on the prevalence of *Borrelia* spp. in questing ticks. Some of the algorithms systematically demonstrated a poorer ability to discern the faunal composition linked to the prevalence.

**Table 1.** Outcome of the modeling algorithms between the vertebrates’ community and the reported prevalence of *Borrelia afzelii* and *Borrelia garinii* in questing nymphs of *Ixodes ricinus* ticks, including clusters 12 to 36, for which there are available data. Cluster (left column) is a consecutive numbering of the unsupervised classification carried out on the target territory as shown in Figure 1(c). The column ‘prevalence’ indicates the averaged reported prevalence of either *B. afzelii* or *B. garinii* together with the number of reported surveys in that cluster in parentheses. Each other column indicates the percent of correct classification of the prevalence of *Borrelia* in questing ticks by the regression and classification algorithms, separately for the two pathogens tested

Cluster	Prevalence: *B. afzelii*	Neural network	Random forest	Gradient boosting	AdaBoost	Prevalence: *B. garinii*	Neural network	Random forest	Gradient boosting	AdaBoost
12	10.55 (3)	Only 3 surveys				5.00 (3)	Only 3 surveys			
13	11.18 (17)	0.625	0.481	1	0.997	5.38 (13)	0.607	0.464	1	1
14	16.58 (14)	0.666	0.338	1	0.947	6.02 (14)	0.816	0.359	1	1
15	14.1 (26)	0.522	0.564	1	0.999	6.83 (26)	0.837	0.639	1	0.995
17	9.21 (11)	0.613	0.761	1	0.999	10.5 (11)	0.985	0.927	1	1
18	8.93 (32)	0.778	0.589	1	0.993	8.46 (32)	0.801	0.737	1	0.998
19	3.62 (10)	1	0.601	1	1	14.03 (10)	0.899	0.855	1	0.998
20	**10.56 (44)**	**0.811**	**0.791**	**1**	**0.988**	**18.61 (44)**	**0.562**	**0.691**	**1**	**0.998**
22	13.45 (9)	1	0.377	1	1	8.83 (9)	1	0.597	1	1
23	7.04 (8)	0.994	0.778	1	0.999	2.67 (8)	1	0.743	1	1
27	3.81 (8)	1	0.426	1	1	8.94 (8)	0.999	0.422	1	1
28	1.09 (5)	0.999	0.133	1	0.555	5.58 (5)	0.994	0.186	1	1
29	21.45 (8)	1	0.487	1	1	20.19 (8)	1	0.543	1	0.989
31	6.18 (8)	1	0.887	1	1	1.06 (8)	0.999	0.777	1	1
32	**22.39 (64)**	**0.443**	**0.655**	**0.999**	**0.999**	15.5 (64)	0.598	0.716	1	0.994
33	0 (1)	Only 1 survey				1.4 (1)	Only 1 survey			
34	12.53 (7)	1	0.25	1	1	8.4 (7)	1	0.455	1	1
35	11.13 (33)	0.866	0.685	1	0.982	**7.21 (33)**	**0.941**	**0.713**	**1**	**0.998**
36	23.08 (11)	0.890	0.448	1	0.998	12.17 (11)	0.998	0.535	1	1

**Figure 7.**
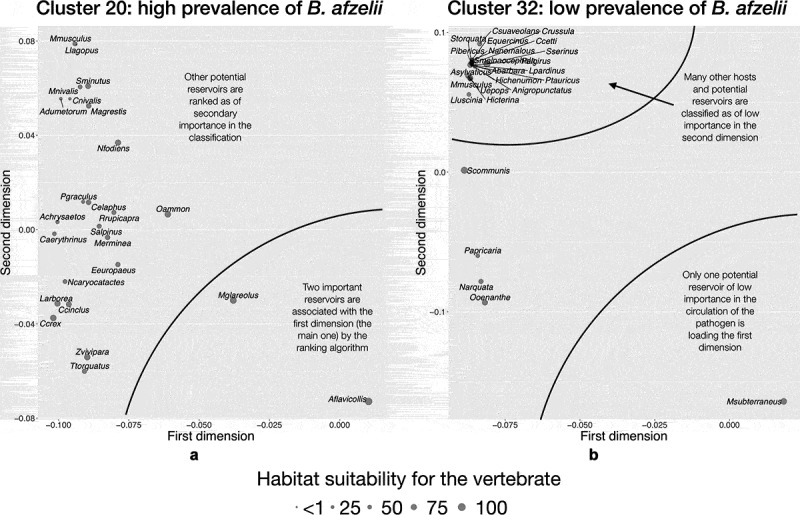
The indicator community of vertebrates projected on the reduced space in two areas of the target territory reporting different rates of infection by *B. afzelii* in questing nymphs of *I. ricinus.*

**Figure 8.**
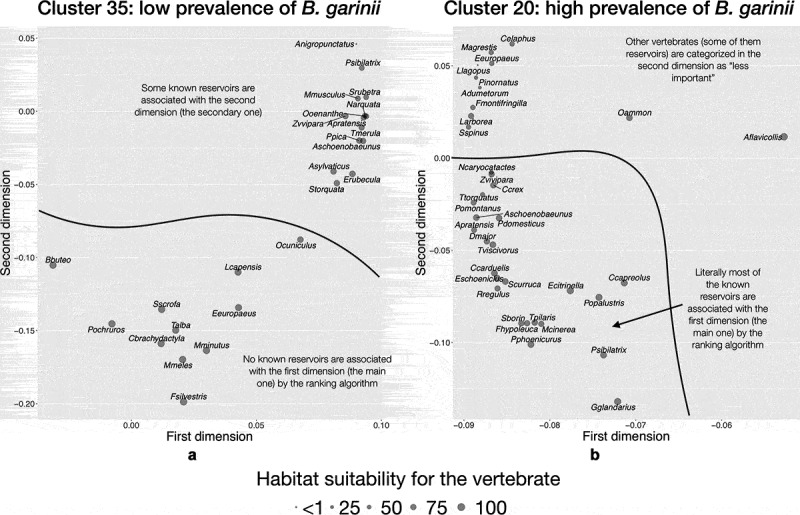
The indicator community of vertebrates projected on the reduced space in two areas of the target territory reporting different rates of infection by *B. garinii* in questing nymphs of *I. ricinus.*

Modeling results for the clusters selected as of high or low prevalence (bold typeface in Table 1) clearly supports the fact that there is an indicator community of vertebrates that results in good correlations with infection rates by *Borrelia* spp. in *I. ricinus*. Gradient Booster provided the best algorithm, with an R^2^ value of more than 0.9 for each tested condition. Figures 7 and 8 expand the ecological explanation of the proof-of-concept and summarize the resulting communities of vertebrates for each condition (cluster+pathogen), separated in the reduced space of principal components after the ranking algorithm. Although these results came from pure modeling, there is agreement between the species included as most/less prominent in each indicator community, and the observed field rates of prevalence of *Borrelia* spp.

Regarding *B. afzelii* (Figure 7(a)), sites belonging to cluster 20 (high prevalence of *Borrelia* reported) have as most contributing vertebrates the Yellow-necked mouse (*Apodemus flavicollis*) and the Bank vole (*Myodes glareolus*). Some Insectivora, ungulates like the Red deer (*Cervus elaphus*), the Chamois (*Rupicapra rupicapra*) and the Mountain sheep (*Ovis ammon*), carnivores, and a few birds are shown as secondary components of the indicator community in that cluster. However, the community defining sites of cluster 32 (low prevalence) is poorer and dominated by the European pine vole (*Microtus subterraneus*), and other species of rodents (i.e., *Mus* spp., *Apodemus* spp.), and Insectivora (i.e., *Crocidura* spp.) that appear distant from the main set of dominant species and closely grouped among them (Figure 7(b)). There is a strong joint occurrence of these secondary species with a low contribution to the reported prevalence of the pathogen in the tick.

A different indicator community was detected for *B. garinii* (Figure 8). Sites reporting low-to-medium prevalence (cluster 35, Figure 8(a)) have several species of mammals, which rank prominently as the main species driving the results of modeling. The only bird among these dominant species is the short-toed treecreeper (*Certhia brachydactyla*). Other species of birds, which are reservoirs of the bacterium, form a community that ranks second in the cluster definition (top of the chart), and that group together, out of the main group of dominant vertebrates. Sites belonging to cluster 20 (highly reported infection rates by *B. garinii* in questing ticks, Figure 8B) are dominated by birds (which are the reservoirs of the pathogen). Some ungulates and Insectivora are part of the community. It is of interest to note that the Chamois, *Capreolus capreolus*, is detected by the modeling algorithms as a prominent part of the indicator community (bottom of the chart) but the Red deer, *Cervus elaphus*, (top of the chart) is not.

Summarizing, the indicator community of sites with high prevalence of *B. afzelii* has a large component of Rodentia and Insectivora (its reservoirs), with birds located in secondary positions. An inverse situation has been detected at sites with low-medium prevalence by the pathogen. Clusters with high prevalence of *B. garinii* are dominated by their reservoir birds, with a significant increase in rodents in clusters in which prevalence is smaller. Carnivora and Ungulata are always secondary members of the indicator community because of their role as tick feeders, contributing to the population of ticks, but not reservoirs.

## Discussion

We demonstrated that a bioregionalization of the western Palearctic can be built with an epidemiological focus on tick-borne pathogens, based on the distribution and abundance of hundreds of species of vertebrates and the contact rates with the tick *I. ricinus*. The classification of modeled distribution maps resulted in clusters reflecting specific combinations of vertebrates and different contact rates with the tick vector. This could provide a strict determination of the impact of changing climate conditions on the predicted distribution of both the tick and the vertebrates, and thus the contact rates and the resulting epidemiological consequences. Previous approaches have aimed at evaluating the background behind the prevalence of *Borrelia* spp. in ticks, considering only abiotic features (Estrada-Peña et al., 2011). The effects of the variability of vertebrates on the infection rates of a tick-transmitted pathogen have never been addressed at anything beyond the regional scale, based on the gold standard based on field or laboratory protocols calculating the prevalence of questing nymphs. However, this study proposed a statistical procedure that establishes the impact of the communities of vertebrates on the infection rates of *Borrelia spp*. in questing *I. ricinus* nymphs, and that is well correlated with the recorded situation in the target territory [22]. This study is a proposal, open to major improvements, that pinpoints an area to be addressed also for other tick-borne pathogens.

The relative importance of several vertebrates in the epidemiology of Lyme borreliosis in Europe has been addressed in literally dozens of studies: not only the key role of rodents or birds as reservoirs of some species [i.e., 16, 41, 42] but also the dual role played by some taxa like large ungulates on the amplification/dilution of the pathogen [i.e., 43, 44]. In the USA, research has been partly focused on the life history traits of different vertebrates, aiming to find a correlate of their contributions to the infection rates of ticks by *B. burgdorferi* s.l. 45 The list of references above is far from complete but provides an appraisal of open debates on the topic. To note, the evaluation of the effect of the vertebrates’ communities on infection rates by *Borrelia* spp. in questing ticks has already been proposed by Mysterud et al. (2019a) but using field experiments.

Normally, only a few species of hosts in foci of tick-transmitted pathogens are studied in the field, most likely due to the impressive logistical issues involved in such surveys or because of the difficulty in collecting scarce vertebrates or protected species [46]. One of the major issues in conducting separate modeling of individual species of organisms is that they may interact in different ways [47,48]. The strategy ‘predict first, then cluster’ as adhered to here, seems to be a good method when modeling groups of co-occurring organisms [20]. Interactions among species are already included in datasets from which the predicted maps are derived: if two or more species compete for a resource and one ends up displaced by the competition phenomena, a lack of records of the affected species will be noticed when the competing species is present.

We observed that higher contact rates of *I. ricinus* with the vertebrates are well correlated for sites in which the phylogenetic diversity of vertebrates is high. This is an important finding since the phylogenetic diversity of an area could be important when species differ in their contribution to the support of the populations of ticks and pathogens. Thus, the phylogenetic composition rather than the list of species in an ecosystem could be of particular importance for understanding the always complex epidemiology of tick-borne pathogens. According to Webb et al. [49], the interpretation is that ‘the more distantly related two species are, the greater the likelihood that they differ ecologically’ (summarized by Cadotte et al. [50]). At sites in which phylogenetic diversity is high, the tick has literally dozens of vertebrates to feed upon since every site in the environmental niche of vertebrates is suitable for the tick. We state that the system, under these conditions, may be highly redundant: the absence of a few key species is replaced by the presence of others that could not be significant under a different community composition.

The concept of indicator species has deep ecological roots in multivariate statistics (Legendre et al. [51]). According to Legendre and De Cáceres [52], a species is ‘an indicator of a group of sites if the indicator value of the species is the highest for that group of sites and is statistically significant at a preselected significance level.’ Our study was based on that concept (revisited by De Cáceres et al. [53]) but aimed to pinpoint indicator *communities* of vertebrates instead of single species. These indicator communities are expected to change in both space and time because of their intrinsic requirements, the availability of resources, and the occasional replacement of species due to trends of climate [54,55]. We previously demonstrated that there are highly significant statistical differences in the infection rates of ticks among sites, for both *B. afzelii* and *B. garinii* 56. However, climate shapes the occurrence of vertebrates and delineates the gradients of contact of the co-occurring species with the vector. This is the intricate niche epidemiology of *Borrelia* spp. that has been already elaborated, using a network analysis [21].

We acknowledge the gaps in this study. Some issues may affect the calculations, such as the sample size of surveyed questing ticks and reported prevalence, the season of the year when the survey was done, local vegetation conditions or landscape fragmentation, or even the method for detecting *the bacterium* (e.g., either pools of ticks or specimens processed individually). Our study also ignored the specific contributions of individual vertebrates’ species to the feeding of the tick or transmission of the pathogen. This is an important point since these data are commonly obtained in field or laboratory protocols [11,57]. A method to evaluate the individual importance of each vertebrate in the circulation of *Borrelia*, aimed to replace the field surveys, and that is based on the entire network of relationships among vertebrates and the tick has been already proposed [21]. This procedure corresponds well with the identification of keystone taxa in ecological studies [58]. Since we are looking for indicator communities, the centrality of the network derived from the matrix of interactions among partners looks like the obvious value to reflect the tick preferences for a host [following 58]. However, no studies have yet linked both field-derived and network-derived data given the paucity of data for many vertebrates. We consider that the validation of the network approach is a necessary step before taking a deeper dive into the reservoir capacity of tick-borne pathogens by vertebrates. The key concept is, ‘how does the combination of several vertebrates affect transmission rates to a single vector?’ At least for *Borrelia* spp., factors allowing speciation are commonly linked to the phylogeny of the reservoir 59,60–63) which fully supports our selection of species and comments on reservoirs.

Results are consistent with the epidemiology of the pathogen, circulating only among some of the vertebrates that feed the tick vector. It is nevertheless of interest to note that the ranking of the vertebrates of each indicator community matches the current knowledge of the most common reservoirs of tested species of *Borrelia*, and points to the dual role played by Carnivora or Ungulata [12]. Most importantly, variability of the communities, in either species composition or abundance, is detected as key factors shaping infection rates in ticks, even if recognized reservoirs are present, but ranked as less important in the community. We think that results are compatible with the knowledge of the ecology of *Borrelia* spp. and their reservoirs. There is still considerable room for improvement of the methods, linking network approaches with spatial modeling and ranking algorithms as well as the basic assumptions, but results are encouraging.

It did not escape our attention that the highest infection rates of both species of *Borrelia* in the three selected groups of clusters have always been found in a more fragmented area (compare, i.e. the spatial fragmentation of clusters in Figure 6). The effect has not been conceptualized in our models since we focused on a purely biological approach regarding the community composition of vertebrates, and because the scale of work would not allow to capture these fine differences. However, the effect has been mentioned in the literature on the topic and even pinpointed as one of the most important effects of preliminary assessments on the distribution of *Borrelia* spp. in Europe 56. On a small scale, the importance of landscape structure has been pointed out as affecting prevalence of some species of *Borrelia*, most probably because of the impact on the diversity of vertebrates’ communities [64]. The scale of our study cannot outline or reject these field studies, but this is an open field that deserves interesting findings when compared with the probable relative rarefaction of some key vertebrates.

We aimed for an ecologically sound and radically different approaches to explain the infection rates of a tick-borne pathogen in the vectors: could a bioregionalization including contact rates among ticks and vertebrates be correlated with the prevalence of *Borrelia* spp. in ticks? Our analysis added an extra dimension that may be of interest to the study of the dynamics of tick-borne pathogens. We anticipate that a wide-open field of research remains ahead of this view: just to cite the example of Tick-borne encephalitis (TBE) that is also transmitted by the same tick and observes a puzzling pattern of distribution, seasonality and (re)emergence of foci [i.e., 65]. We hope this approach can provide innovative ways to approximate the complex epidemiology of many tick-borne pathogens using a synthetic background [42,44,56,66–68].

## Supplementary Material

Supplemental Material
