# Supplementary material for: Is composition of vertebrates an indicator of the prevalence of tick-borne pathogens?
Source: Infect Ecol Epidemiol. 2022 Jan 10;12(1):2025647. doi: 10.1080/20008686.2022.2025647 (PMC8757609; doi:10.1080/20008686.2022.2025647)
Supplement: Supplemental Material [file ZIEE_A_2025647_SM3067.zip › Supplementary Material/Suplemental Material 1.docx]

The phylogenetic tree of the set of species of vertebrates used in this study

Species of vertebrates found in cluster #3 and used as hosts by *Ixodes ricinus* (blue dots).

Species of vertebrates found in cluster #4 and used as hosts by *Ixodes ricinus* (blue dots).

Species of vertebrates found in cluster #5 and used as hosts by *Ixodes ricinus* (blue dots).

Species of vertebrates found in cluster #6 and used as hosts by *Ixodes ricinus* (blue dots).

Species of vertebrates found in cluster #7 and used as hosts by *Ixodes ricinus* (blue dots).

Species of vertebrates found in cluster #8 and used as hosts by *Ixodes ricinus* (blue dots).

Species of vertebrates found in cluster #9 and used as hosts by *Ixodes ricinus* (blue dots).

Species of vertebrates found in cluster #10 and used as hosts by *Ixodes ricinus* (blue dots).

Species of vertebrates found in cluster #11 and used as hosts by *Ixodes ricinus* (blue dots).

Species of vertebrates found in cluster #12 and used as hosts by *Ixodes ricinus* (blue dots).

Species of vertebrates found in cluster #13 and used as hosts by *Ixodes ricinus* (blue dots).

Species of vertebrates found in cluster #14 and used as hosts by *Ixodes ricinus* (blue dots).

Species of vertebrates found in cluster #15 and used as hosts by *Ixodes ricinus* (blue dots).

Species of vertebrates found in cluster #16 and used as hosts by *Ixodes ricinus* (blue dots).

Species of vertebrates found in cluster #17 and used as hosts by *Ixodes ricinus* (blue dots).

Species of vertebrates found in cluster #18 and used as hosts by *Ixodes ricinus* (blue dots).

Species of vertebrates found in cluster #19 and used as hosts by *Ixodes ricinus* (blue dots).

Species of vertebrates found in cluster #20 and used as hosts by *Ixodes ricinus* (blue dots).

Species of vertebrates found in cluster #21 and used as hosts by *Ixodes ricinus* (blue dots).

Species of vertebrates found in cluster #22 and used as hosts by *Ixodes ricinus* (blue dots).

Species of vertebrates found in cluster #23 and used as hosts by *Ixodes ricinus* (blue dots).

Species of vertebrates found in cluster #25 and used as hosts by *Ixodes ricinus* (blue dots).

Species of vertebrates found in cluster #26 and used as hosts by *Ixodes ricinus* (blue dots).

Species of vertebrates found in cluster #27 and used as hosts by *Ixodes ricinus* (blue dots).

Species of vertebrates found in cluster #28 and used as hosts by *Ixodes ricinus* (blue dots).

Species of vertebrates found in cluster #29 and used as hosts by *Ixodes ricinus* (blue dots).

Species of vertebrates found in cluster #30 and used as hosts by *Ixodes ricinus* (blue dots).

Species of vertebrates found in cluster #30 and used as hosts by *Ixodes ricinus* (blue dots).

Species of vertebrates found in cluster #31 and used as hosts by *Ixodes ricinus* (blue dots).

Species of vertebrates found in cluster #32 and used as hosts by *Ixodes ricinus* (blue dots).

Species of vertebrates found in cluster #33 and used as hosts by *Ixodes ricinus* (blue dots).

Species of vertebrates found in cluster #34 and used as hosts by *Ixodes ricinus* (blue dots).

Species of vertebrates found in cluster #35 and used as hosts by *Ixodes ricinus* (blue dots).

Species of vertebrates found in cluster #36 and used as hosts by *Ixodes ricinus* (blue dots).
