# Supplementary material for: Is composition of vertebrates an indicator of the prevalence of tick-borne pathogens?
Source: Infect Ecol Epidemiol. 2022 Jan 10;12(1):2025647. doi: 10.1080/20008686.2022.2025647 (PMC8757609; doi:10.1080/20008686.2022.2025647)
Supplement: Supplemental Material [file ZIEE_A_2025647_SM3067.zip › Supplementary Material/Suplemental material 2.docx]

Species of vertebrates found in clusters #4, #5, #7 and #8 and used as hosts by *Ixodes ricinus* (blue dots), together with a map display the spatial distribution of the target clusters. Information as a short table is included about phylogenetic diversity (PD) and species richness (SR) in each cluster

Species of vertebrates found in clusters #3, #6, #16, #22, #29, #31, #36 and used as hosts by *Ixodes ricinus* (blue dots), together with a map display the spatial distribution of the target clusters. Information as a short table is included about phylogenetic diversity (PD) and species richness (SR) in each cluster.

Species of vertebrates found in clusters #13, #20, #21, #23, #25, #26, #28, #30, and #32 and used as hosts by *Ixodes ricinus* (blue dots), together with a map display the spatial distribution of the target clusters. Information as a short table is included about phylogenetic diversity (PD) and species richness (SR) in each cluster.

Species of vertebrates found in clusters #9, #12, #14, #15, #17, #18, #27, #33, #34, and #35 and used as hosts by *Ixodes ricinus* (blue dots), together with a map display the spatial distribution of the target clusters. Information as a short table is included about phylogenetic diversity (PD) and species richness (SR) in each cluster.
